# Transversus abdominis plane block for lumboperitoneal shunt surgery in idiopathic normal pressure hydrocephalus: a case report

**DOI:** 10.1093/jscr/rjab123

**Published:** 2021-04-19

**Authors:** Jinduck Cho, Jinseok Yeo, Sung Hyun Chang, Sang-Youl Yoon, Seong-Hyun Park, Kyunghun Kang, Chi-Hun Kim, Myong Hun Hahm, Eunhee Park, Ki-Su Park

**Affiliations:** Department of Anesthesia and Pain Medicine, School of Medicine, Kyungpook National University, Daegu, Republic of Korea; Department of Anesthesia and Pain Medicine, School of Medicine, Kyungpook National University, Daegu, Republic of Korea; Department of Neurosurgery, School of Medicine, Kyungpook National University, Daegu, Republic of Korea; Department of Neurosurgery, School of Medicine, Kyungpook National University, Daegu, Republic of Korea; Department of Neurosurgery, School of Medicine, Kyungpook National University, Daegu, Republic of Korea; Department of Neurology, School of Medicine, Kyungpook National University, Daegu, Republic of Korea; Department of Neurology, School of Medicine, Kyungpook National University, Daegu, Republic of Korea; Department of Radiology, School of Medicine, Kyungpook National University, Daegu, Republic of Korea; Department of Physical and Rehabilitation Medicine, Kyungpook National University Medical Center, Daegu, Republic of Korea; Department of Neurosurgery, School of Medicine, Kyungpook National University, Daegu, Republic of Korea

## Abstract

The transversus abdominis plane (TAP) block is an ideal pain control method used in surgeries that require abdominal wall incisions through the injection of an anesthetic solution into the plane between the internal oblique muscle and transversus abdominis muscle. Herein, we report an 83-year-old man who was diagnosed with idiopathic normal pressure hydrocephalus (iNPH) and underwent lumboperitoneal shunt surgery (LPS). The TAP block was performed before LPS, and the numerical rating scale for pain was 0 at day 1 after the surgery. The patient was discharged early at day 3 after surgery despite the patient being extremely old, as he reported quick relief from the postoperative abdominal pain. The TAP block can hence be considered for use before LPS in elderly patients with iNPH.

## INTRODUCTION

The transversus abdominis plane (TAP) block is an interfascial plane block to provide analgesia to the anterolateral abdominal wall. It anesthetizes thoracolumbar nerves from the T6 to L1. It is performed by injection of a local anesthetic solution between the internal oblique muscle (IOM) and transversus abdominis muscle (TAM) [1]. Lumboperitoneal shunt surgery (LPS) is an alternative treatment for cerebrospinal fluid (CSF) diversion [2–4]. The path of a distal catheter passes across the paraspinal region, flank and anterolateral abdomen under the skin and is inserted into the peritoneal cavity [5]. Due to the similarity between the path of a catheter in LPS and a sensory dermatome affected by a TAP block, a TAP block before LPS is likely to relieve postoperative wound pain. Here, we present a preoperative TAP block application before LPS in an advanced aged patient with idiopathic normal pressure hydrocephalus (iNPH).

**Figure 1:**
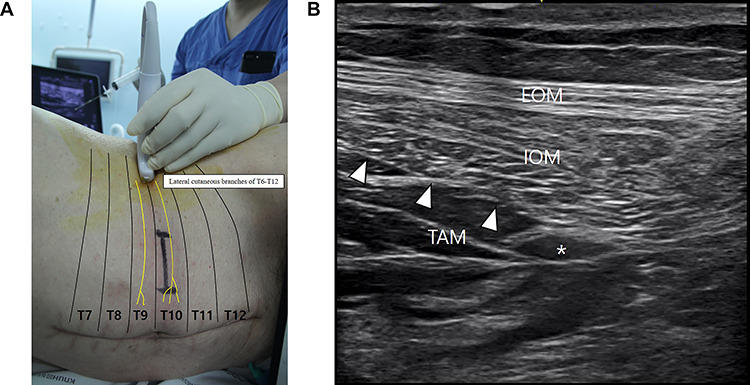
The TAP block. (**A**) The linear transducer was placed transversely on the lateral abdominal wall at the left midaxillary line between the subcostal margin and the iliac crest. (**B**) Ultrasound view showing a local anesthetic solution (asterisk) injected and spread into the plane between the IOM and TAM. *EOM*: external oblique muscle, *IOM*: internal oblique muscle, *TAM*: transversus abdominis muscle.

**Figure 2:**
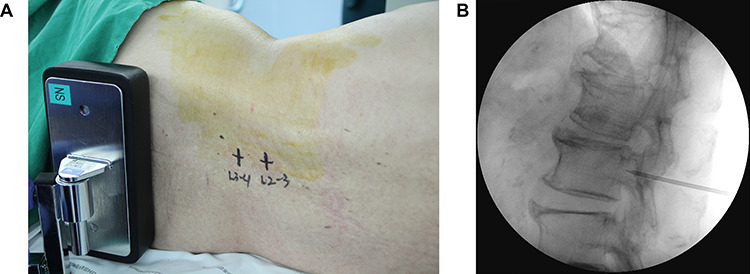
The lumboperitoneal shunt surgery. (**A**) Initial dermal marking made on the patient’s back at the interspinal space of L2–3 and L3–4. (**B**) Tuohy needle was advanced toward the target near the lower border of the L3 vertebral body under fluoroscopic guidance.

## CASE REPORT

An 83-year-old-man presented with a 3-year history of gait impairment. His brain MRI revealed a higher Evans’ index (>0.3), ventricle enlargement and a typical disproportionately enlarged subarachnoid space hydrocephalus sign [6]. iNPH was confirmed, indicating consideration for shunt surgery. However, the patient was afraid of brain surgery and was worried about abdominal pain because he already suffered abdominal pain from colon cancer surgery in the past 6 months. Therefore, a preoperative TAP block and LPS were simultaneously performed.

After induction of general anesthesia using lidocaine (40 mg), propofol (80 mg) and rocuronium (50 mg) and maintenance with desflurane, the patient was placed in a right lateral decubitus position. The linear transducer (L 11-3® [3–11 MHz], Konica Minolta, Japan) of the ultrasound (SONIMAGE HS1®, Konica Minolta, Japan) was placed transversely on the lateral abdominal wall at the left midaxillary line between the subcostal margin and the iliac crest (Fig. 1A). Using an in-plane approach, the positions of the IOM and TAM were spontaneously examined. A 22-gauge, 80-mm Tuohy-type needle (Epidural needle®, Hakko Co., Ltd, Japan) was inserted in the fascia layer between the IOM and TAM to inject 20 ml of 0.25% ropivacaine solution (Fig. 1B).

Initial dermal marking was made on the patient’s back at the interspinal space of L2–3 and L3–4 (Fig. 2A). The Tuohy needle was inserted at 1 cm lateral to the midline of the spinous process and on the interspinal space of L2–3 and then advanced toward the spinal canal under fluoroscopic guidance (Fig. 2B). When the Tuohy needle reached the CSF space, the spinal catheter was inserted. A strata adjustable programmable valve (Medtronic Neurologic Technologies, Medtronic Inc., Goleta, CA, USA) was connected to the spinal catheter. After that, the distal catheter was connected to the valve and then inserted into the abdominal cavity in a conventional manner.

After the operation, the numerical rating scale for pain at the surgical site using the visual analog scale was assessed for 2 h (1 h postoperation, 3 points; 2 h postoperation, 4 points) at the ward. Then, the patient started to ambulate without pain (0 points) 1 day after the surgery and was discharged early after 3 days.

## DISCUSSION

Since Rafi’s 2001 description, TAP blocks have become one of the most commonly applied truncal blocks [7–9]. Until now, the TAP block has been an ideal pain control method used in surgeries that require abdominal wall incisions, such as appendectomy, cesarean section, hysterectomy, posterior prostatectomy, laparoscopic cholecystectomy, hernia surgery and colon resection [9–11]. Although a case of TAP block in ventriculoperitoneal shunt surgery was reported, there has been no report on the usage of TAP block for LPS despite the path of LPS being sufficiently covered by TAP block [12].

LPS is a surgical procedure in which the tip of a proximal catheter is inserted into the lumbar cistern, while the distal catheter is tunneled under the skin and into the abdominal cavity. The Japanese Society of Normal Pressure Hydrocephalus investigated the safety and efficacy of the LPS for iNPH and suggested that LPS could be beneficial for patients with iNPH and can act as a first-line of treatment option for this disease. Moreover, Kawahara *et al*. [13] performed LPS using local anesthesia on elderly patients and suggested that LPS is possible with local anesthesia. Our case experience suggests the possibility of using a TAP block for LPS in terms of postoperative quick pain reduction. Moreover, if the TAP block is combined with local anesthesia for LPS, it would become an ideal treatment method for iNPH in advanced aged patients (≥80 years) and in those at high risk from general anesthesia.

In conclusion, postoperative abdominal pain can be quickly relieved in LPS through the use of a TAP block in elderly patients with iNPH. This approach is expected to allow the possibility of shunt surgery using local anesthesia in the future.
